# Nephron-Sparing Strategies for Tumor-Bearing Horseshoe Kidney: From Surgeries to Pharmaceuticals

**DOI:** 10.3390/ph19101660

**Published:** 2026-10-21

**Authors:** Meng-Zhen He, Hui Zhou

**Affiliations:** 1 Department of Urology, Tongji Hospital, Tongji Medical College, Huazhong University of Science & Technology, Wuhan 430030, China; hhhmmmzzz0806@163.com; 2 Department of Nursing, Tongji Hospital, Tongji Medical College, Huazhong University of Science & Technology, Wuhan 430030, China; 3 Hubei Provincial Clinical Research Center for Minimally Invasive Treatment of Urology, Wuhan 430030, China

**Keywords:** horseshoe kidney, renal cell carcinoma, partial nephrectomy, organ preservation, ischemia-reperfusion injury

## Abstract

**Background/Objectives:** Horseshoe kidney (HSK) is a type of congenital renal fusion anomaly with extreme anatomic complexity. The malrotation of kidneys and ureters and malformation and altered isthmus and vascular supply may boost several renal and extrarenal complications. Organ preservation of tumor-bearing HSK is rather difficult and only available in a small minority of patients. **Methods/Results:** This review introduces new advances for nephron-sparing surgical strategies for tumor-bearing HSK, such as super-selective artery clamping, ultrasound-guided renal artery balloon catheter occluded hybrid partial nephrectomy, and focal therapies. It also puts forward novel pharmaceuticals for kidney function preservation to alleviate ischemia-reperfusion injury and medications for neoadjuvant therapies. **Conclusions:** HSK possesses unique etiology, complex anatomic characteristics, and hidden complications. Renal function preservation is extremely important for tumor-bearing HSK. For long-term high-quality lifespan, precise preoperative planning, surgeon’s versatility with multiple surgical techniques, and novel pharmaceuticals to avoid IRI are promising strategies for nephron-sparing surgery.

## 1. Introduction

Horseshoe kidney (HSK), also called *ren arcuatus* in Latin, is the most common congenital renal fusion anomaly, with an estimated incidence of 1 in 500 individuals and a male predominance of 2:1. The well-known U-shaped structure, like that of a horseshoe, is usually created by a parenchymal or fibrous isthmus connecting the inferior poles (superior poles in less than 10% of cases of HSK variants) of two kidneys [1,2]. HSK, not a newly discovered disease, was firstly described in 1522 by Berengario da Carpi in *Isagogae breves* (Introduction to Anatomy) [3]. Tumors are rarely encountered in the context of HSK, making the optimal management of tumor-bearing HSK an intractable challenge for urologists. There is a scarcity of comprehensive reviews which systematically address the pathobiological characteristics, clinical features and surgical procedures regarding renal fusion and ectopia. By searching the full-text articles in English in the PubMed database with key search terms like horseshoe kidney, nephrectomy, and nephron-sparing, our study aims to elucidate the nephron-sparing options for tumors in HSK by critically appraising the literature surrounding the basic research and clinical advances related to HSK.

## 2. Embryology and Pathogenesis of HSK

The embryogenesis and histogenesis of the collecting system and kidneys have been progressively gaining importance to explain the pathogenesis of HSKs [2]. The functioning kidney and collecting system are derived from the metanephric mesenchymal blastema and ureteric bud, respectively, meeting at the upper sacral region (S1 and S2) to form the metanephros under proper signaling through reciprocal induction. Each metanephros then ascends from the sacral region to lumbar area below the rib cage during gestational weeks 6 to 9 of embryological development [3,4]. Aberrant metanephros migration, mechanical factors, disrupted molecular signaling or genetic and environmental factors can collectively contribute to HSK development.

### 2.1. Close Proximity of the Two Metanephros

The most prominent theory of renal fusion is that the two metanephros come into close apposition during ascent, resulting from the developmental impedance by an arterial fork such as the inferior mesenteric artery (IMA). The distance between the two metanephros and renal fusion can be caused by aberrant cell migration across the sagittal plane, mutation-associated renal hyperplasia or dysplasia in the medial segment of the metanephros, or cell lineage segregation and migration due to aberrant morphogenetic signaling from abdominal aorta [5].

### 2.2. Mechanical Factors

The isthmus most often consists of parenchymal tissue,; less commonly, it is predominantly composed of fibrous tissue from ectopic mesenchymal tissue, and patients with given HSK variants are susceptible to having concomitant vertebral anomalies. Mechanical fusion either from rotation of the caudal embryo or lateral flexion of the trunk is contemplated for explaining the relevance of asymmetric HSK to congenital vertebral diseases.

### 2.3. Disrupted Genetic and Molecular Signaling, Environmental Factors

Disruptions of certain molecular signaling such as notochord signaling and the sonic hedgehog (Shh) pathway can induce kidney fusion, as shown by recent animal models. The secreted protein Shh mediates signaling activities of notochord and floor plate, which determine the position of the metanephric kidney; inactivation of Shh signaling can lead to failed renal separation and migration, accordingly [6]. Additionally, WT1 gene mutations may be responsible for HSK because WT1 is indispensable for metanephric mesenchyme and renal cell proliferation. In consonance with this hypothesis, patients with HSK anomalies are inclined to suffer from Wilms tumor [4,7]. HSK is also associated with genetic mutations and chromosomal disorders like Turner syndrome. Meanwhile, environmental exposures to toxic substances like thalidomide can also involve the genesis of renal ectopia [8].

## 3. Clinical Features, Vascular Pattern, and Complications of HSK

**Figure 1:**
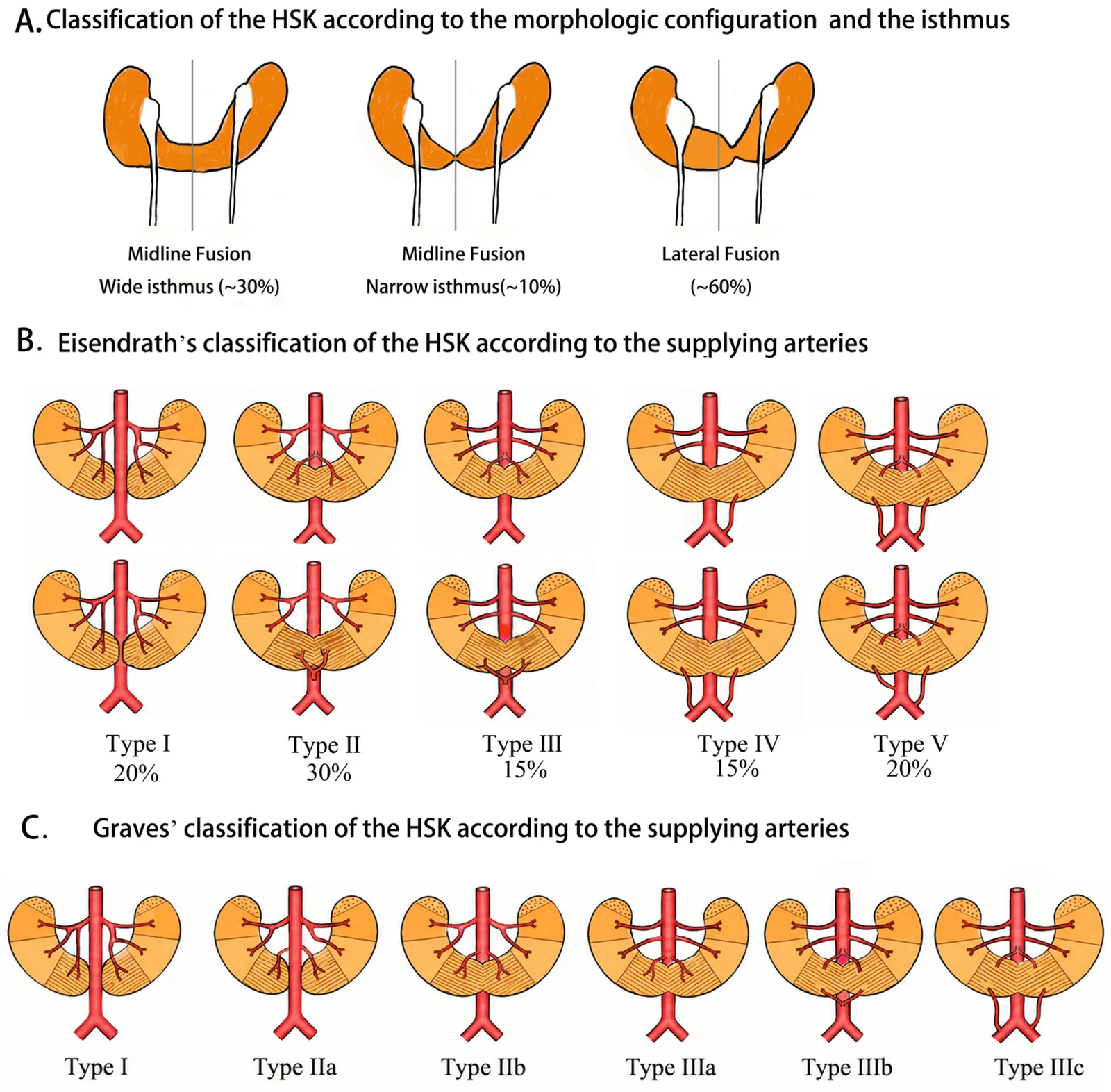
Classification of horseshoe kidney (HSK). (**A**) Classification of HSK according to the morphologic configuration (midline fusion or lateral fusion) and the isthmus (narrow or wide). (**B**) Eisendrath’s classification of HSK; the percentages of each type are also described. (**C**) Graves’ classification of HSK according to the supplying arteries.

HSK is most frequently positioned anterior to the aorta at spinal segments L3–L4, while the ureters descend from the kidney anterior to the isthmus, with the potential of kinking or obstruction by renal arteries. The morphological configurations and components of the isthmus are variable: midline fusion accounts for only 42% of all cases, and lateral fusion is more common; the isthmus can be narrow or wide, with components of pure parenchymal tissue, fibrous/mesenchymal tissue or mixed tissues (Figure 1A) [4].

The blood supply of HSK shows considerable variability and high unpredictability. Some individuals may exhibit more than five or six renal arteries originating from the aorta, inferior mesenteric artery, iliac or sacral arteries [9]. Eisendrath and Graves even classified HSK according to the distribution of the supplying arteries (Figure 1B,C, and Table 1) [1,10]. Unlike separated kidneys, the control of HSK vessels at the hilum may be insufficient to control bleeding during surgery because arteries derived from the IMA, lower level of aorta, or iliac/sacral artery may be neglected. Accessory isthmus arteries are prevalent in HSK cases, and the isthmus is often supplied by separate arteries from IMA, anterior aorta or common iliac arteries [11]. Previous theory indicates that the IMA can “hold back” the isthmus at the L3 level; however, only 40% of cases comply with this assumption, and another 60% of cases have isthmus lying at L4 below the IMA or even in the pelvis [12]. The abnormal renal vasculature coinciding with HSK variants prompts questions about whether the medial fusion of metanephros is the cause or is the consequence of altered vascular supply. The renal arteries tend to branch off their parental vessels below the IMA and are slim in diameter [8]. Moreover, the venous drainage of HSK is equally complex, with multiple veins returning to inferior vena cava, iliac veins, or azygos veins.

The abnormal position (ectopia) of HSK results in malrotation of kidneys and ureters. Malformation and altered vascular supply may further boost several renal and extrarenal complications, although most individuals with HSK are asymptomatic and diagnosed incidentally with image examination. Sharp ureteric angles at the ureteropelvic junction, abnormal collecting system across the isthmus, and the compression of atypical vascular supply predispose to renal complications including ureteropelvic junction obstruction (UPJO), hydronephrosis, nephrolithiasis, recurrent urinary tract infection (UTI) and neoplasms [13]. Patients with HSK are susceptible to chronic kidney disease (CKD) or even end-stage renal disease (ESRD), mandating chronic hemodialysis [14]. Furthermore, patients with HSK have a proclivity to suffer from open wounds and blunt-force renal trauma owing to the superficial midline position of the isthmus and absence of partial protection from the rib cage [15,16]. They are also at high risk of intraoperative complications due to anatomical variation, limited mobilization of kidneys and complex vasculature organization involving accessory vascular branches [17]. In addition, HSK is frequently associated with extrarenal diseases and congenital syndromes including gastrointestinal tract and cardiovascular diseases, genitourinary defects such as vesicoureteral reflux, vertebral anomalies and hereditary diseases such as Turner syndrome and Down syndrome.

## 4. Characteristics of Tumors in HSK

**Figure 2:**
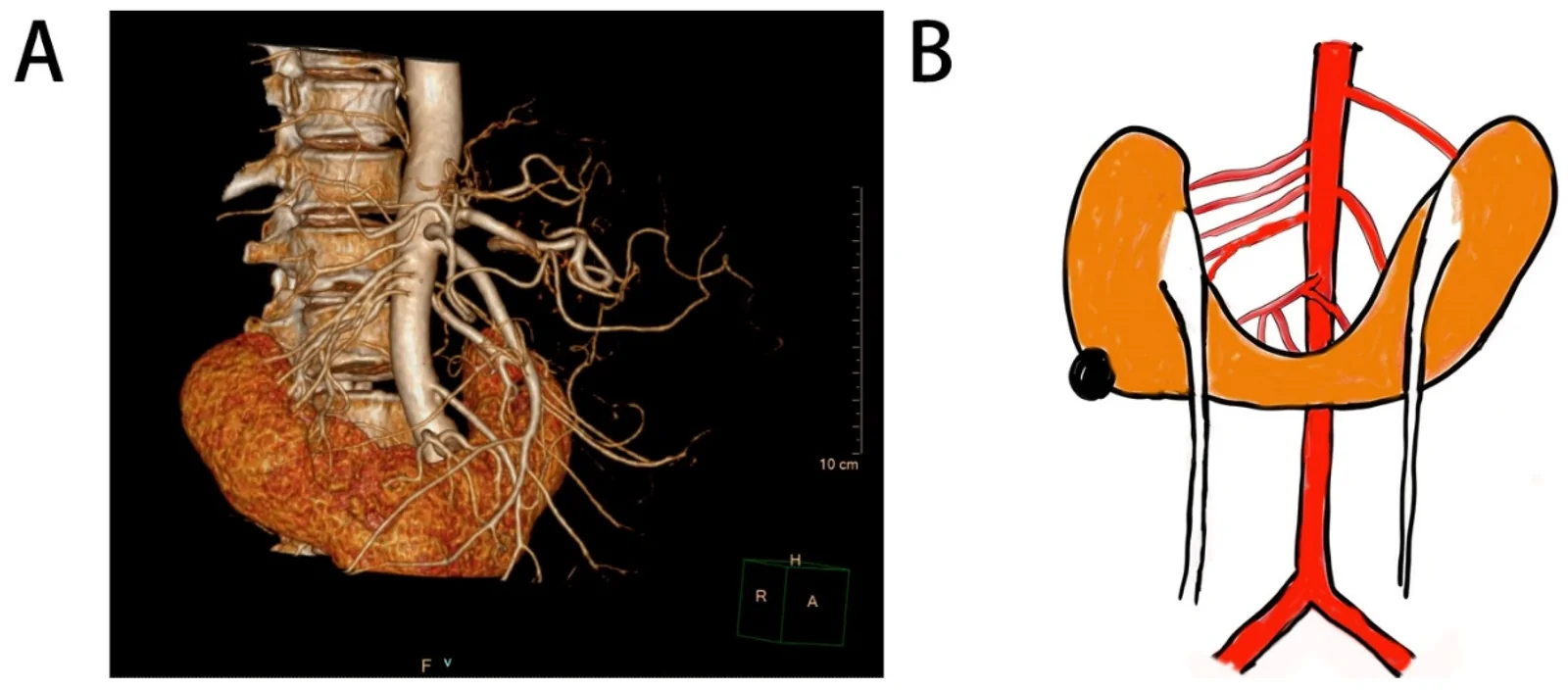
A CT angiogram (**A**) and diagrammatic map (**B**) of a cancer-bearing HSK with multiple arteries (four arteries for right kidney and two arteries for left kidney from aorta, and one artery from the aorta under the IMA for isthmus). The colors of arteries, kidney cortex, renal pelvic, and tumor are red, orange, white, and black, respectively.

Neoplasms associated with HSK exhibit great variation in histological subtypes and morphological spectrum. No definite evidence supports that HSK facilitates the tumorigenesis of renal tumors, although positive correlations have been found between HSK and both Wilms tumors and renal carcinoid tumors. Figure 2 presents a CT angiogram and diagrammatic map of a cancer-bearing HSK with multiple arteries.

### 4.1. Renal Cell Carcinoma (RCC)

Due to the sparse data of case series and absence of persuasive cross-sectional surveys, whether the HSK anomaly increases the possibility of RCC remains unclear, although RCC accounts for nearly 50% of all tumors in the HSK context [18,19,20,21,22,23,24,25]. Similarly to the general population, clear cell RCC, papillary RCC, and chromophobe RCC are among the most frequent histological subtypes observed in HSK patients [26,27,28,29,30]. Additionally, some genetic mutations, chromosomal aberrations or chronic complications may contribute to the occurrence of RCC, such as Xp11.2 translocation [31] and chronic hemodialysis for CKD [32].

### 4.2. Renal Neuroendocrine Tumors (NETs)

NETs are rare neoplasms originating from foci of metaplastic or teratomatous epithelium of the kidney that include well-differentiated renal carcinoid tumors, large cell neuroendocrine carcinoma (LCNEC), and small cell carcinoma (SCC) [33]. Renal carcinoid tumors are a common type of genitourinary carcinoids, in addition to testicular or ovarian ones, and the current literature implies a remarkable susceptibility of HSK patients to renal carcinoid tumors [33,34,35,36,37,38]. Most cases of renal carcinoid tumor are asymptomatic; only a small proportion of patients present with pain in the abdominal/flank region, hematuria or neuroendocrine syndromes [35,36]. The biological behavior of carcinoid tumor is heterogeneous, and most carcinoids in the kidney are relatively more indolent than RCC from the standpoint of cellular proliferation rate [39,40]. Most tumors are observed at an early age without gender predilection; patients with renal carcinoid tumors tend to have neoplasms larger than 4 cm, and distant metastases at initial diagnosis are prevalent especially for large tumors. Renal carcinoid tumors seem to possess unique cytogenetic aberrations compared to carcinoids from other anatomical sites, including loss of heterozygosity on chromosome 3p21 and aberration on chromosome 13 [39,40,41]. Immunohistochemical staining for neuroendocrine-related markers includes synaptophysin, chromogranin A, and CD56. Somatostatin receptor scintigraphy or F-DOPA PET/CT may facilitate the precise diagnosis and positioning of renal NETs [35,42].

### 4.3. Wilms Tumors

Wilms tumors, also called nephroblastomas, are a common pediatric malignancy in kidney relevant to Trisomy 18 or Xp22.11 duplication. HSK is associated with a higher risk of developing Wilms tumors partially owing to the above-mentioned shared germline alterations [43,44].

### 4.4. Other Occasional Carcinomas in HSK

Urothelial cancers are extremely rare in HSK, and the standard surgical approach, including nephroureterectomy (concurrent isthmusectomy) and bladder cuff excision to eradicate all suspicious transitional tissues, is difficult in the context of HSK [45,46]. Renal medullary carcinoma is an extremely aggressive subtype of RCC, characterized by sickle cell traits and deletion of the SMARCB1 gene; only one case of medullary carcinoma in HSK has been discovered far, during an autopsy [47]. Clear cell sarcoma of kidney (CCSK) is also extraordinary rare in the HSK context, harboring epidermal growth factor receptor internal tandem duplication (ITD) rather than conventional YWHAE-NUTM2B/E fusion or BCOR-ITD [48]. Mucinous adenocarcinoma of the renal pelvis, commonly identified by painless hematuria or cystic mass, has been reported only in sparse cases of HSK [49,50,51,52,53].

### 4.5. Benign Tumors

Nephron-sparing surgical procedures may be feasible for some benign tumors simultaneously with HSK, like hemangioma and oncocytoma [54,55,56], emphasizing the importance of preoperative biopsy and active surveillance for non-aggressive, low-risk and localized masses suspected of cancer.

### 4.6. Bilateral/Multiple Tumors, Isthmus Tumors

Despite their low probability, bilateral/multiple tumors or isthmus tumors in HSK pose overwhelming therapeutically challenges under the organ-sparing dilemma [56,57,58]. Controlling the feeding arteries of synchronous tumors or isthmus tumors might increase the duration of ischemia and the risk of intraoperative complications. Multidisciplinary discussion should give priority to active surveillance, focal therapies and non-surgical systematic therapies.

## 5. Nephron-Sparing Modalities for Tumor-Bearing HSK

HSK patients are susceptible to multiple complications and renal insufficiency. Nephron-sparing surgery (NSS), which permits optimum preservation of renal function, imposes huge challenge for urologists, not to mention the surgery in the tumor-bearing HSK scenario. The overarching goal for tumor-bearing HSK management is to achieve optimal oncologic control (complete tumor excision with negative margins) while maximizing preservation of renal function and minimizing complications.

### 5.1. Preoperative Planning

In the HSK context, not all tumor-feeding arteries are located at the hilum; the configuration of the renal pedicle is also variable. Controlling the vessels at the hilum alone may be insufficient to prevent excessive bleeding due to accidental omission of arteries from common iliac arteries or different levels of abdominal aorta [11]. Great vascular complexity and diversity necessitate detailed preoperative diagnostic procedures (typically with CT angiography, magnetic resonance imaging) to visualize the precise anatomy (e.g., accessory isthmus arteries, surrounding structures) and avoid catastrophic intraoperative bleeding. The accurate visualization of horseshoe kidney vasculature and meticulous planning would undoubtedly facilitate various abdominal and pelvic surgeries, in particular, organ-preserving surgery of tumor enucleation. In addition to conventional imaging of the vasculature with CT angiograms, MR angiography, and interventional renal arteriography, three-dimensional volume rendering technology to generate interactive anatomical models or 3D virtual reconstruction has been reported to facilitate the surgical planning [59,60,61,62,63].

### 5.2. Treatment Modalities for Tumor-Bearing HSK

Nephron-sparing surgery, primarily partial nephrectomy, is the standard of care for treating localized renal tumors when technically feasible [64,65]. As for HSK, relatively healthy moieties are also at high risk of developing CKD, and optimum preservation of renal parenchyma requires even more emphasis. However, before the advent of laparoscopic techniques, open surgery with heminephrectomy and isthmusectomy (division of isthmus) was the mainstream option; NSS accounted for only a small share [66]. Unparalleled tactile feedback and maneuverability permit full exposure of the vascular supply and adjacent organs, lymph node dissection, thrombectomy for inferior vena cave/renal vein tumor thrombus, and pyeloplasty/ureteroplasty for contralateral moieties are also feasible under the condition of open surgery. Therefore, while minimally invasive options have emerged as a paradigm shift to promote rapid recovery, open surgery remains a benchmark for challenging situations like huge, advanced, bilateral or hilar tumors and tumors in the isthmus.

Laparoscopic surgeries emerged as promising alternatives for renal tumors in the 1990s. The surgical approach can employ the transperitoneal or retroperitoneal route; radical nephrectomy or NSS are both practicable to meet distinct needs. Hand-assisted and single-port approaches are utilized in appropriate cases [46,67]. Laparoscopic partial nephrectomy (LPN) has become the dominant approach at many medical centers, making it possible for the urologist to preserve renal function with smaller incisions, reduced postoperative pain and shorter recovery, while requiring advanced technical skills and posing limitations in suturing and maneuverability [62,68,69,70]. With the assistance of 3D visualization, wristed instruments, and improved ergonomics in robotic laparoscopic platforms, robot-assisted partial nephrectomy (RAPN) facilitates precise reconstruction and reduces warm ischemia time to make NSS feasible for a broader range of tumor complexities like completely endophytic tumors or hilar tumors [71].

While doubts remain for oncological outcomes of treating aggressive malignancies with focal therapy, patients with extreme anatomic complexity such as HSK and high anesthetic risk such as comorbid cardiovascular/pulmonary conditions might gain the maximum benefit. Ablative techniques are tissue-destructive NSS alternatives for small tumors. In some pilot studies, thermal ablation (radiofrequency ablation, CT-guided microwave), percutaneous cryoablation, and stereotactic ablative body radiotherapy (SABR) have emerged as promising adjuvant strategies for HSK [58]. However, perirenal adhesion or retroperitoneal fibrosis are also possible after focal therapies. For low-volume and low-risk tumors, focal therapies are also applicable if patients prefer radical measures rather than biopsy or active surveillance. Additionally, extracorporeal bench surgery combined with renal autotransplantation can be used for complex renal surgery; however, the inherent anatomic complexity may limit its usage in HSK.

### 5.3. Technical Strategies for NSS in Tumor-Bearing HSK

The basic process of NSS comprises tumor exposure and positioning, clamping of all tumor-feeding arteries (ischemia), resection of the tumor, suturing the remaining kidney (defects, collecting system and bleeding vessels) [72]. The main strategies for decreasing renal parenchyma/function loss are vascular control, ischemia management, and tumor excision methods during operation.

The management of renal blood flow during tumor excision is critical to prevent blood loss, attenuate ischemia-reperfusion injury (IRI), and protect long-term renal function. Versatility with NSS approaches helps minimize warm ischemia time (clamping time at body temperature). However, the allowable ischemia time (25 to 30 min) is not enough for complex tumors requiring prolonged reconstruction. Cold ischemia (dropping the kidney temperature with ice slush or irrigation solution) can dramatically slow metabolism and extend the allowable ischemia time [73]. However, some techniques can only block the arteries supplying the area of resection to avoid global renal ischemia. Super-selective artery clamping with preoperative 3D-reconstruction planning allows the surgeon to identify and clamp only the tumor-feeding segmental arteries [61,74]. Preoperative super-selective artery embolization can also be implemented for the purpose of early vascular control. Li Heng and colleagues developed a new surgical approach named ultrasound-guided renal artery balloon catheter occluded hybrid partial nephrectomy (UBo-HPN) for main or segmental renal artery occlusion, especially suitable for recurrent renal tumors or perirenal adhesion ones [75,76,77]. Although this technique has not been utilized for HSK, the difficulty of dissecting renal arteries in HSK may bring its superiority into full play. In addition, controlled hypotensive anesthesia to lower mean arterial pressure can help reduce bleeding without clamping. Similarly, performing the initial renorrhaphy stitch before tumor resection helps complete the tumor bed suturing under controlled bleeding without clamping [78,79].

Tumor excision and suturing techniques also play important roles in reducing parenchymal loss. Standard sharp excision requires cancer resection with a rim of normal parenchyma using cold scissors or a blade. For ccRCC with natural pseudocapsule, tumor enucleation can maximize parenchymal preservation and reduce ischemia time, without compromising oncologic outcomes when a negative margin is achieved. Intraoperative indocyanine green fluorescence navigation and three-dimension printing reconstruction can also reduce unnecessary resection of normal parenchyma for cases with ill-defined margins [29,30,70]. The argon beam coagulator (ABC) allows for optimal tumor enucleation and coagulation on the tumor bed, and shortens the warm ischemia times significantly compared to internal renorrhaphy; even off-clamp procedures are possible in T1a tumors [80,81]. Recently, Wang et al. engineered a blood-triggered, dual-cross-linked adhesive hydrogel loaded with reactive oxygen species-responsive (ROS-responsive) GW7647 liposomes, which could rapidly solidify to achieve effective hemostasis, acting as a tension-free sealant to replace or reduce suturing [82].

Regardless of strategy, the “Trifecta” of ideal outcomes is achieving negative oncologic margins, minimal renal functional decline, and the absence of urologic complications. The most common complication of tumor resection in HSK is postoperative hemorrhage, which can be managed by renal arteriography and embolization [83]. However, urine extravasation/leakage and persistent urinoma formation are also not uncommon; a double-J stent or even secondary surgery may be needed.

### 5.4. Pharmaceuticals for Kidney Function Preservation

Novel and improved techniques for partial nephrectomy are only creeping forward, while pharmaceuticals to mitigate IRI during renal transplantation and other organ preservation surgery have been showing rapid progress in recent decades. Nephron-sparing surgeries could inevitably incur immediate nephron loss and initial ischemic tubular necrosis upon IRI, while prolonged ischemic stress may further retard the functional and structural recovery from acute kidney injury (AKI), inducing the AKI-CKD transition [84]. High mobility group box 1 (HMGB1) signaling, the STING pathway, and inflammation/redox signaling are widely studied, and targeting these pathways is promising for preventing AKI and eventual CKD [85].

The administration of a conventional drug, iron sucrose, protects against renal IRI in kidney transplant patients by enhancing mitochondrial respiratory complex assembly [86]. A traditional compound, Shenkangling, whose active ingredients are catalpol and loganin, alleviates renal fibrosis induced by renal IRI by mitigating mitochondrial damage through modulation of the STING signaling pathway [87]. Additionally, several nano-medicines help relieve acute and long-term renal impairment by regulating ROS generation, calcium overload, or inflammatory pathways. Adhesive hydrogel loaded with GW7647 liposomes can suppress lipid peroxidation and block ferroptosis by activating the PPARα/Nrf2/GPX4-SLC7A11 signaling axis with the aid of an ischemic microenvironment [82]. Jiang et al. developed a pH-activatable pre-nanozyme which promoted H_2_S gas release for endo-exogenous regulation of oxidative stress in AKI [88]. This remedy also may be promising for HSK cases. Potential limitations of these nano-medicines include high medical cost, potential toxicity like hepatorenal toxicity and immunological reactions, and poor stability and biocompatibility. Consequently, rigorous clinical trials are warranted before their clinical application. Unfortunately, these studies are mainly conducted in animal models; no clinical trials which evaluate the renal protective effects of drugs after nephron-sparing surgeries are available (Table 2). However, for localized-aggressive cancers or high-volume tumors, neoadjuvant targeted or immunological therapy may facilitate the complete and precise resection of tumors and renal function preservation.

## 6. Conclusions and Perspectives

**Figure 3:**
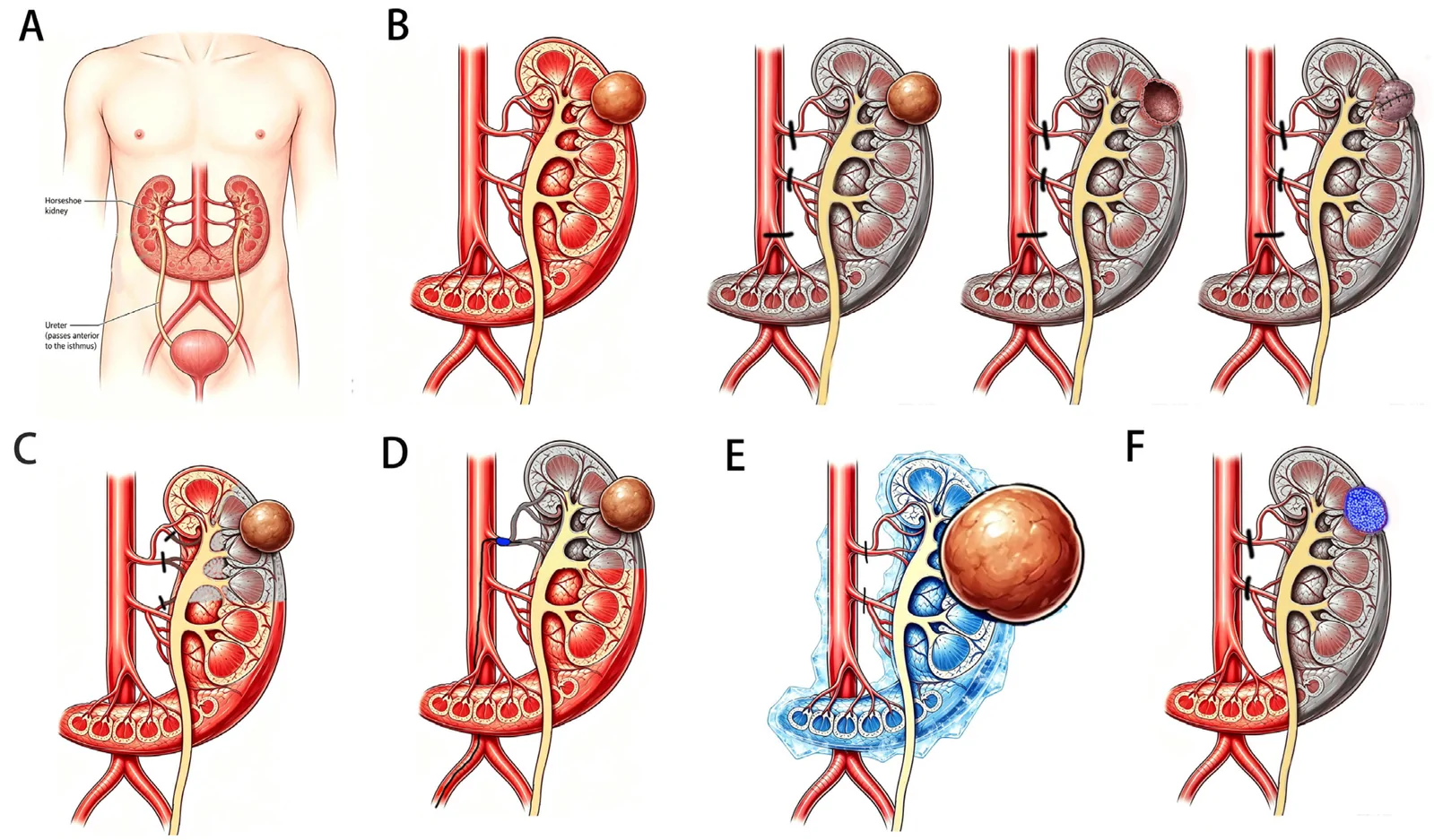
Some novel techniques and therapies for the organ preservation of tumor-bearing HSK. (**A**) Representative graph of HSK and its spatial relationship with aorta and ureter. (**B**) Conventional methods of partial nephrectomy for tumor-bearing HSK: tumor exposure and positioning, clamping of all tumor-feeding arteries, resection of the tumor, suturing the remaining kidney. (**C**) Super-selective arterial clamping with preoperative 3D-reconstruction planning allows surgeons to identify and clamp only the tumor-feeding segmental arteries. (**D**) UBo-HPN, which can occlude main or segmental renal arteries without the need for arterial dissection, is especially suitable for recurrent renal or perirenal adhesion tumors. (**E**) Cold ischemia with ice slush or irrigation solution can dramatically slow metabolism and extend the allowable ischemia time, and is especially suitable for huge or complex cancers. (**F**) A blood-triggered, dual-cross-linked adhesive hydrogel loaded with ROS-responsive liposomes can rapidly solidify to achieve effective hemostasis, acting as a tension-free sealant to replace or reduce suturing.

Tumor-bearing horseshoe kidney is a rare but challenging disease. Organ protection is of paramount importance for patients to prolong their overall survival and improve their quality of life. Based on the anatomic characteristics and the mechanism for alleviating IRI, several techniques and pharmaceuticals developed for conventional partial nephrectomy can be remodeled to meet the demand of nephron-sparing in the context of tumor-bearing HSK (Figure 3). Considering the extended ischemic time, multiple but slim arteries, and high risk of adjacent organ injury, some techniques like cold ischemia and super-selective artery clamping may be extremely suitable for HSK. However, advances in surgical skills have been dragging on despite efforts. Fortunately, clinical trials for drugs, signaling and therapeutic targets for kidney injury, and progress in materials science may shed light on remedies for alleviating acute kidney injury and chronic renal fibrosis. Also, in crossed fused renal ectopia (CFRE) and other congenital developmental malformations with great anatomical complexity, the treatment options may be utilized to treat tumors with maximal preservation of functional renal parenchyma.

The anatomic complexity of HSK and characteristics of tumors (higher proportion of neuroendocrine tumors) determine that there will be differences in nephron-sparing strategies, especially in pharmaceutical treatment. Multiple and complex renal artery supplies and isthmus will increase the difficulty of arterial clamping and parenchymal suturing. As a result, off-clamp procedures and suture-free procedures may be more important for tumors in HSK. Additionally, patients with HSK are more prone to suffer from CKD. Novel pharmaceuticals and hydrogels to alleviate IRI or lower surgical complexity are urgently needed in treating tumor-bearing HSK. Furthermore, patients with tumor-bearing HSK will benefit more from targeted therapies for neuroendocrine tumors.

This review has some shortcomings to be addressed. Given the extreme rarity of tumor-bearing HSKs, the study inherently relies on low-level clinical evidence or preclinical research, such as small-sample retrospective case series and individual case reports. Although some studies conducted in conventional kidney or other organs are included, differences in organ preservation strategies may be unavoidable, and extrapolation may be doubtful.

In conclusion, HSK possesses unique etiology, complex anatomic characteristics, and hidden complications. For patients with tumor-bearing HSK, renal function preservation is extremely important for a long-term, high-quality lifespan. Precise preoperative planning, surgeon versatility with multiple surgical techniques, and novel pharmaceuticals to avoid IRI are promising strategies for nephron-sparing surgery.

## Figures and Tables

**Table 1:** Eisendrath’s and Graves’ classification of the HSK.

	Type	Isthmus	Arteries from Aorta	Arteries from Iliac
Eisendrath	I	Two kidneys not joined across the midline	One pair of bilateral arteries (HSK side)	None
	II	Fusion	One pair of bilateral arteries (HSK side) One unilateral branch from the aorta (isthmus)	None
	III	Fusion	Two pairs of bilateral renal arteries (HSK side) One unilateral branch from the aorta (isthmus)	None
	IV	Fusion	Two pairs of bilateral renal arteries (HSK side)	One or more branches from the iliac arteries (isthmus)
	V	Fusion	Multiple renal arteries originating from either the aorta or iliac arteries (HSK side)	One or two branches from the iliac arteries (isthmus)
Graves	I	Two kidneys not joined across the midline	One pair of bilateral arteries (HSK side)	None
	II a	Two kidneys not joined across the midline	Two pairs of bilateral renal arteries (HSK side)	None
	II b	Fusion	One pair of bilateral arteries (HSK side) Two lower-segment arteries form a common trunk	None
	II c	Fusion	Posterior branches of the artery have arisen earlier, and outside the renal substance	None
	III a	Fusion	The middle and the lower-segment arteries arise directly from the aorta	None
	III b	Fusion	The lower poles of both kidneys receive twin vessels from the aorta, both above and below the isthmus.	None
	III c	Fusion	The lower poles of both kidneys receive twin vessels from the aorta, both above and below the isthmus.	The posterior branch of the lower-segment artery arises from the bifurcation of the aorta or the common iliac arteries

**Table 2:** Pharmacological strategies for renal function preservation.

Drugs	Ref	Targets	Types of Models Used	Efficacy & Side Effects
Glycyrrhizic acid; Ethyl pyruvate	85	AKI-CKD transition; HMGB1	Mouse model of persistent kidney hypoxia	Prevents AKI-CKD transition
Iron sucrose	86	Mitochondrial respiratory complex assembly	Kidney transplant patients Mouse renal IR injury model	Positive correlation between elevated iron levels and improved renal allograft outcomes in patients
Shenkangling (Catalpol/loganin)	87	Mitochondrial damage, STING signaling pathway	Unilateral Renal ischemia-reperfusion injury mouse model, TGF-β-induced HK-2 cell fibrosis model	Alleviates renal IRI-induced renal fibrosis
GW7647 liposomes	82	PPARα/Nrf2/GPX4-SLC7A11 signaling axis	Surgical model with SD rats	Rapid and noncompressive hemostasis, rapid tissue regeneration and functional recovery
Ultra-small Platinum sulfur cluster (Pt_5.65_S)	88	Oxidative damage and inflammation, H_2_S gas, reactive oxygen/nitrogen species, Nrf2	Mouse models of kidney ischemia-reperfusion injury and cisplatin-induced AKI	Mitigates inflammatory diseases such as AKI; remarkably low biological toxicity

## Data Availability

No new data were created or analyzed in this study. Data sharing is not applicable to this article.

## Abbreviations

The following abbreviations are used in this manuscript:

HSK	Horseshoe Kidney
IMA	Inferior Mesenteric Artery
RCC	Renal Cell Carcinoma
NSS	Nephron-Sparing Surgery
PN	Partial Nephrectomy
LPN	Laparoscopic Partial Nephrectomy
NETs	Neuroendocrine Tumors
IRI	Ischemic Reperfusion Injury
AKI	Acute Kidney Injury
CKD	Chronic Kidney Disease
